# IVIg Treatment Reduces Catalytic Antibody Titers of Renal Transplanted Patients

**DOI:** 10.1371/journal.pone.0070731

**Published:** 2013-08-15

**Authors:** Ankit Mahendra, Ivan Peyron, Cécile Dollinger, Laurent Gilardin, Meenu Sharma, Bharath Wootla, Séverine Padiolleau-Lefevre, Alain Friboulet, Didier Boquet, Christophe Legendre, Srinivas V. Kaveri, Olivier Thaunat, Sébastien Lacroix-Desmazes

**Affiliations:** 1 Centre de Recherche des Cordeliers, Université Pierre et Marie Curie, Unité Mixte de Recherche S 872, Paris, France; 2 Université Paris Descartes, Unité Mixte de Recherche S 872, Paris, France; 3 Institut national de la santé et de la recherche médicale U872, Paris, France; 4 Hospices Civils de Lyon, Hôpital Edouard Herriot, Service de Transplantation, Néphrologie et Immunologie Clinique, Lyon, France; 5 Génie Enzymatique et Cellulaire, Unité Mixte de Recherche 6022 Centre National de la Recherche Scientifique. Université de Technologie de Compiègne, Compiègne, France; 6 Institut de biologie et de technologies de Saclay, Service de Pharmacologie et d'Immunoanalyse, Laboratoire d'Ingénierie des Anticorps pour la Santé, Commissariat à l'énergie atomique, Saclay, Gif-sur-Yvette, France; 7 Service de Transplantation Rénale et de Soins Intensifs, Hôpital Necker, Assistance publique-hôpitaux de Paris, Paris, France; 8 Laboratoire international associé Institut national de la santé et de la recherche médicale, Paris, France; 9 Indian Council of Medical Research, New Delhi, India; 10 Institut national de la santé et de la recherche médicale, U851, Lyon, France; 11 Université de Lyon, Lyon, France; Universidade de Sao Paulo, Brazil

## Abstract

Catalytic antibodies are immunoglobulins endowed with enzymatic activity. Catalytic IgG has been reported in several human autoimmune and inflammatory diseases. In particular, low levels of catalytic IgG have been proposed as a prognostic marker for chronic allograft rejection in patients undergoing kidney transplant. Kidney allograft is a treatment of choice for patients with end-stage renal failure. Intravenous immunoglobulins, a therapeutic pool of human IgG, is used in patients with donor-specific antibodies, alone or in conjunction with other immunosuppressive treatments, to desensitize the patients and prevent the development of acute graft rejection. Here, we followed for a period of 24 months the levels of catalytic IgG towards the synthetic peptide Pro-Phe-Arg-methylcoumarinimide in a large cohort of patients undergoing kidney transplantation. Twenty-four percent of the patients received IVIg at the time of transplantation. Our results demonstrate a marked reduction in levels of catalytic antibodies in all patients three months following kidney transplant. The decrease was significantly pronounced in patients receiving adjunct IVIg therapy. The results suggests that prevention of acute graft rejection using intravenous immunoglobulins induces a transient reduction in the levels of catalytic IgG, thus potentially jeopardizing the use of levels of catalytic antibodies as a prognosis marker for chronic allograft nephropathy.

## Introduction

Catalytic antibodies are immunoglobulins that are endowed with enzymatic activity [1]. The first examples of catalytic antibodies were obtained following the active immunization of experimental animals with appropriate immunogens, referred to as transition state analogs [1], [2], [3]. Since then, a series of approaches has been elaborated to generate antibodies with desired enzymatic activities [4], [5], [6]. Antibodies with enzymatic properties however also develop spontaneously *in vivo*. Thus, IgG able to hydrolyze the vasoactive intestinal peptide, DNA, thyroglobulin or pro-coagulant factor VIII have been described in patients with asthma, systemic lupus erythematosus, Hashimoto's thyroiditis and hemophilia A, respectively [7], [8], [9], [10], [11]. Because catalytic antibodies in the human had been reported under pathological conditions, it was long thought that they are endowed with a pathogenic role, or that, at least, they are a hallmark of immune dysregulation and uncontrolled inflammation [12]. However, catalytic antibodies of the IgM, IgG and IgA isotypes have since been reported in normal blood, in the milk of healthy mothers and in saliva [13], [14], [15]. Under physiological conditions, the antigen/substrate specificity of catalytic antibodies is promiscuous [16] and the latter are generally probed using surrogate synthetic peptide substrates [17]. It was proposed that catalytic antibodies may participate in immune homeostasis and in clearance of biological wastes [18], [19], in line with the hypothesis proposed by P Grabar regarding naturally occurring antibodies [20]. Interestingly, we have recently demonstrated a correlation between the increased prevalence of catalytic IgG and positive outcome in several human diseases. Thus, increased levels of IgG capable of hydrolyzing the synthetic tri-peptide substrate for serine proteases - proline-phenylalanine-arginine-methyl-coumarinamide (PFR-MCA), were found at the time of diagnosis in patients who had survived septic shock three weeks later [21]. Similarly, we documented the presence of PFR-MCA-hydrolyzing IgG in the plasma of patients undergoing renal transplant [22]. Low levels of catalytic IgG 3 months following transplantation were predictive of chronic allograft nephropathy (CAN), the main cause for late allograft failure, 2 years down the lane, suggesting that IgG-mediated PFR-MCA hydrolysis may be used as a prognosis marker for CAN in renal-transplanted patients [22].

Accumulating evidence from experimental models [23] and clinical studies [24] suggests that humoral immunity plays a central role in the development of chronic allograft nephropathy (for a recent review see: [25]). Indeed, unlike T cells, which have progressively come under pharmacologic control, the humoral arm of the recipient's immune response remains insufficiently tamed by modern immunosuppressive armamentarium. As a result, anti-donor specific antibodies (DSA) can be detected in the circulation of an increasing proportion of graft recipients with time [26]. Circulating DSA, mostly directed against mismatched HLA molecules, bind to directly accessible allogenic targets expressed by endothelial cells of the graft microvasculature, which triggers the activation of the classical complement pathway and recruits innate immune effectors. Chronic inflammation in turn promotes progressive tissue destruction resulting in irreversible loss of graft function. Some patients, referred to as “sensitized” patients, have preformed DSA, i.e., DSA generated before the transplantation as the result of pregnancies, blood transfusion, and/or previous transplant, and are therefore considered to be at high risk for accelerated chronic rejection [27]. We have recently shown that an intensive day 0 prophylactic immunosuppressive strategy combining intravenous immunoglobulin (IVIg), anti-CD20 and plasmapheresis in this high-risk population is associated with a significant improvement in the long-term function and chronic antibody-mediated rejection rate [28]. IVIg represents a pool of normal human IgG purified from the plasma of several thousands of healthy donors, the use of which has been reported in a number of pathological conditions [29]. The precise mechanism by which IVIg suppresses harmful inflammation has not been definitively established but several studies have reported the decline of pathogenic antibody titers following IVIg administration. No information is currently available on the impact of such treatment on catalytic antibodies.

In the present intermediate study, we followed the levels of catalytic IgG in a large cohort of patients with renal transplant for a period of 24 months post-transplant. The results document higher levels of PFR-MCA-hydrolyzing IgG in patients prior to transplant, as compared with pooled IgG from healthy individuals. A drastic reduction in the levels of catalytic antibodies is observed in all patients three months following kidney transplant. The decrease is significantly more pronounced in patients receiving adjunct IVIg therapy. Taken together, the results suggests that the treatments dedicated at preventing acute graft rejection induce a transient reduction in the levels of catalytic, thus potentially jeopardizing the use of levels of catalytic antibodies as a prognosis marker for CAN.

## Patients and Methods

### Study population

From February 2008 to August 2009, we prospectively collected plasma from consecutive patients 3 months following renal transplant at the Renal Transplantation Department of the Necker Hospital (Paris, France). Plasma was also collected 12 months and 24 months post-transplant. Frozen pre-transplant and pre-treatment plasma samples were retrieved retrospectively when available (n = 59). Clinical characteristics of the patients were collected at the same time-points during physical examination and are depicted in Table 1. Written informed consents were obtained from each patient according to the Declaration of Helsinki. Some sensitized patients had received IVIg at the time of renal transplant. The protocol for IVIg treatment consisted in 2 g per kg body weight Endobulin® (Baxter, Maurepas) over a 96-hr period of time on the day of transplant, on day 21, 42 and 63 after kidney transplant.

**Table 1:** Characteristics of the study population.

		IVIg-treated	No IVIg	P value*
Number of patients		24	76	
Sex - M/F		11/13	39/37	
Age - years†		46.9±3.1 (21–73)	48.8±1.7 (22–83)	ns
Weight – kg		68.0±2.9 (51–109)	66.4±1.7 (39–114)	ns
	ND	0	1	
Previous transplant - %		47.8	7.1	**<0.0001**
	ND	1	6	
Cause of Nephropathy – Nb (%)#				
	Diabetes	1 (4.2)	3 (4.0)	ns
	Vascular	0 (0)	8 (10.5)	ns
	Glomerulopathy	3 (12.5)	19 (25.0)	ns
	Uropathy	10 (41.7)	15 (19.7)	0.056
	Interstitial nephropathy	2 (8.3)	13 (17.1)	ns
	Unknown	8 (33.3)	18 (23.7)	ns
Dialysis time prior to transplant - m†		65.2±9.4 (7–148)	50.2±5.5 (0–214)	0.091
	ND	1	13	
HLA mismatch - score: 1 to 6 (range)		4 (0–5)	3 (0–6)	ns
	ND	0	2	
Anti-HLA1 and anti-HLA2 Abs - %		81.8	48.2	**0.006**
	ND	13	20	
Adjuvant immunotherapy - Nb‡				
	Anti-thymocyte globulins	16	12	
	Basiliximab	9	45	
	Rituximab	7	3	
	Plasmapheresis	2	3	
	None	11		

* Two-tailed Mann-Whitney test; † Mean±SEM (range); ND: Not Documented; ^#^Fisher's exact test.

‡ All patients received Steroids, Cyclosporin, Tacrolimus and/or Mycophenolate Mofetil; one patient received both Basiliximab and anti-thymocyte globulins.

### Ethics statement

The blood samples were taken during the normal follow-up of the patient and since the study did not require additional blood sampling, an approval from an ethics committee was not required under French law according to the article L.1121-1 of the public health code. The article states that: The research organized and performed on human beings in the development of biological knowledge and medical research are permitted under the conditions laid down in this book and are hereinafter referred to by the term “biomedical research”. The article further states that it does not imply under conditions: “For research in which all actions are performed and products used in the usual way, without any additional or unusual diagnostic procedure or surveillance.”

### Plasma collection

Blood was collected in citrate Vacutainer® tubes (BD biosciences), and centrifuged at 1500 rpm for 10 min at 20°C. Plasma was stored in aliquots at −20°C until use.

### Purification of IgG

IgG were isolated from serum by affinity-chromatography on protein G-Sepharose (Amersham Pharmacia Biotech). In brief, IgG was incubated with protein G-Sepharose overnight at 4°C, eluted using 0.2 M glycine-HCl pH 2.8, dialyzed against PBS-0.02% NaN3 overnight at 4°C, and concentrated using Amicon (Millipore). A therapeutic preparation of pooled normal human IgG (intravenous Ig (IVIg); Sandoglobulin®) was used as a source of control IgG. Size-exclusion chromatography of patients' IgG and IVIg was performed on a Superose-12 column (GE Healthcare Europe) equilibrated with urea-containing buffer (50 mM Tris pH 7.7, 8 M urea and 0.02% NaN3), at a flow rate of 0.5 ml/min to exclude potentially contaminating proteases. IgG-containing fractions were then pooled and dialyzed against PBS-0.02% NaN3 for 2 days with four changes in buffer at 4°C, followed by dialysis against catalytic buffer containing 5 mM CaCl2 (pH 7.7) for 1 day with two changes in buffer at 4°C. The purity of IgG preparations was confirmed by SDS-PAGE and immunoblotting under non-reducing conditions. IgG was quantified by Bradford assay. As indicated elsewhere [16], control experiments were performed to demonstrate that the proteolytic activity of the antibodies was intrinsic and did not reflect protease contamination.

### IgG-mediated hydrolysis of PFR-MCA

IgG (66.67 nM) were mixed with 100 µM PFR-MCA (Peptide Institute, Inc.) in 40 µl of catalytic buffer containing 5 mM CaCl2 (pH 7.7) in white 96-well U-bottom plates (Thermo Scientific) and incubated in the dark for 24 h at 37°C. Hydrolysis of the PFR-MCA substrate was determined by the fluorescence of the leaving group (aminomethylcoumarin; λem 465 nm, λex 360 nm) using a spectrofluorometer (GENios; Tecan Trading). Fluorescence values were compared with a standard curve of free MCA and the corresponding quantities of released MCA were computed. At each time point, background release of MCA, measured in wells containing the substrate alone, was subtracted from the value observed in the presence of the Abs. Data are expressed as the quantity of released MCA computed at time 0 subtracted from the quantity of released MCA computed at a given time point per amount of time per amount of IgG.

### Statistics

The statistical comparisons of groups of patients treated with IVIg and of patients not treated with IVIg were performed using the non-parametric Mann-Whitney test, with two-tailed p values.

## Results

### Circulating IgG from renal-transplanted patients hydrolyze PFR-MCA

Plasma was collected prospectively from 100 renal-transplanted patients at 3, 12 and 24 months post-transplant from February 2008 to September 2011. Fifty-nine pre-transplant plasma samples were retrieved retrospectively. Overall, 27 patients were lost during the 24 months of the study period (1 died and 26 lost to follow-up). The characteristics of the cohort are presented in Table 1. Briefly, the cohort included as many men as women, with a mean age of 48.3±1.5 years (mean±SEM; range: 21 to 83). Fifty-four % of the patients were sensitized (not documented in 33 cases). Eighteen had anti-HLA I DSA, 29 anti-HLA II, and 10 both. All the patients were transplanted with a negative CDC cross-match. Fifteen and 2 patients had had one or two previous transplants, respectively. A large majority of the patients were under classical tri-therapy using Tacrolimus, Mycophenolate mofetil and steroids for maintenance of immunosuppression. Fifty-three patients received Basiliximab (including 9 who also received IVIg) and 28 patients received anti-thymocyte globulins (including 16 with IVIg). Ten patients received Rituximab alone (1 case), with Basiliximab (1 case), with anti-thymocyte globulins (7 cases) or with both (1 case). Finally, 18 patients did not receive any induction therapy.

**Figure 1:**
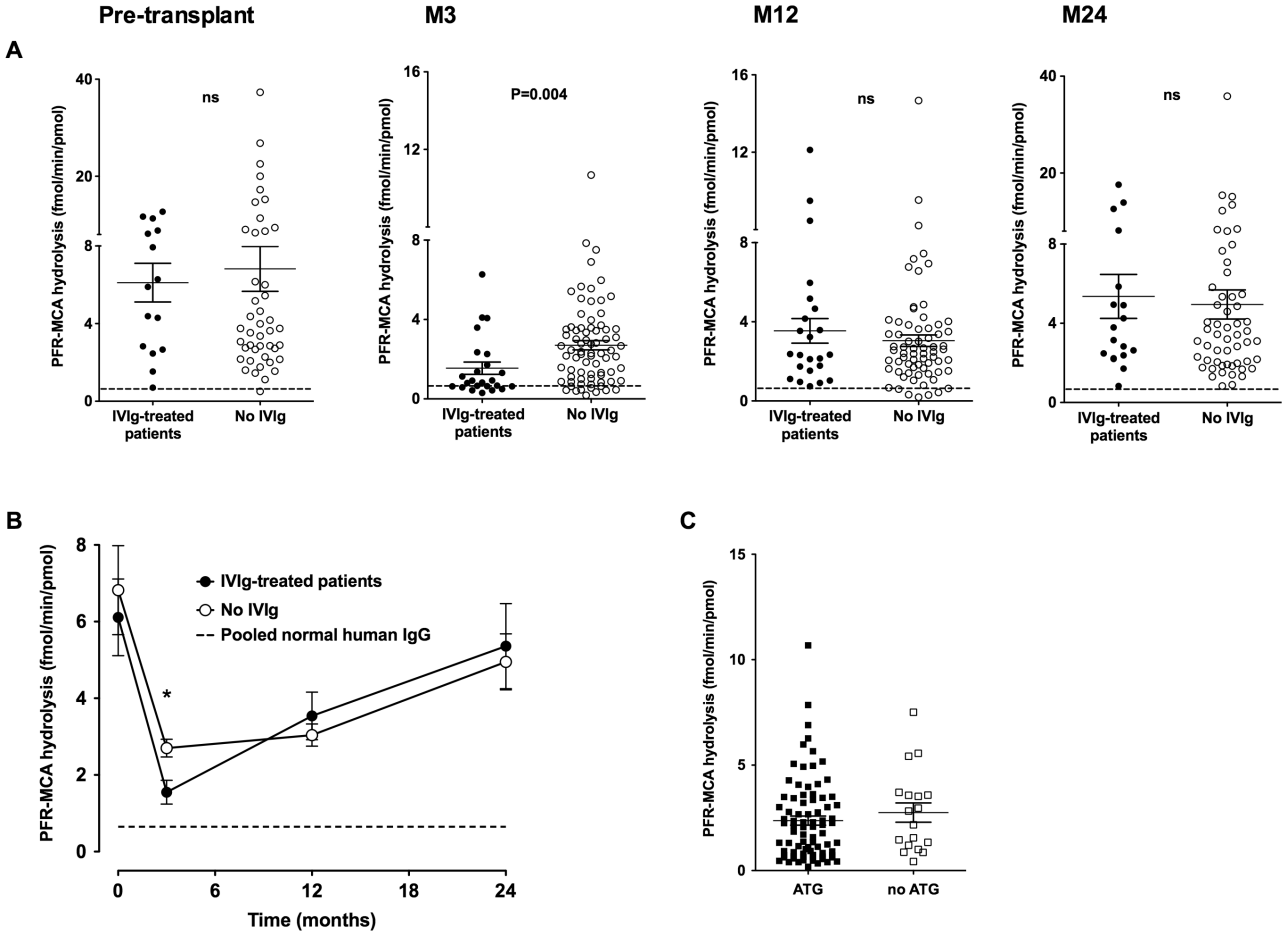
Treatment with IVIg is associated with a transient decrease in levels of PFR-MCA hydrolyzing IgG. IgG was purified from the plasma of patients who received IVIg therapy prior to transplantation (full circles) and from patients who did not received IVIg (empty circles). Plasma had been collected prior to renal transplant (D0) and 3 (M3), 12 (M12) and 24 (M24) months after renal transplant. IgG (66.67 nM) was incubated with PFR-MCA (100 µM), a peptide chromogenic substrate, for 24 hr at 37°C. The amount of hydrolysis was quantified by measuring the fluorescence of the leaving MCA moiety, and is expressed in femtomoles of substrate hydrolyzed per minute per picomoles of IgG. Pooled normal human IgG was used as a control source of IgG. Panel A depicts the raw results as scatter dot plots. Panel B depicts the evolution of the mean ± SEM levels of PFR-MCA-hydrolyzing IgG in the two groups of patients with time (*: P = 0.004). The dotted line represents the hydrolysis of PFR-MCA by normal pooled human IgG (mean of 29 measurements; Coefficient of variation: 0.29). Panel C depicts the levels of PFR-MCA-hydrolyzing IgG in patients treated with anti-thymocyte globulins (ATG, full squares) or not (empty squares), as measured in plasma collected 3 months post-transplantation.

IgG was purified and tested for hydrolysis of the peptide PFR-MCA, a surrogate substrate for catalytic antibodies with serine protease-like activity [17]. The absence of contamination of the IgG samples by adventitious proteases was ensured by the use of a double-step purification procedure that involves a step of purification based on affinity and a step of purification based on protein size under denaturing conditions. Incubation of patients' IgG with PFR-MCA resulted in hydrolysis of the peptide and release of the fluorescent MCA moiety. The released fluorescence allowed for the calculation of rates of hydrolysis. Hydrolysis of PFR-MCA was dose and time-dependent (data not shown). Pooled IgG from healthy individuals demonstrated a marginal hydrolysis of PFR-MCA with an activity of 0.65±0.03 fmol/min per pmol (mean±SEM for 29 repeats). Irrespective of the time-point considered, IgG from renal-transplanted patients demonstrated significantly higher hydrolysis rates of PFR-MCA than pooled IgG from healthy individuals (Figure 1). The levels of PFR-MCA hydrolyzing IgG were extremely heterogeneous prior to transplantation, with a mean activity of 6.6±0.9 fmol/min/pmol (mean±SEM; coefficient of variation: 1.04, Figure 1). They decreased during the first three months that followed renal transplant to reach 2.4±0.2 fmol/min/pmol, and increased gradually during the 21 following months (3.2±0.3 and 5.1±0.6 fmol/min/pmol at 12 and 24 months post-transplant, respectively).

### Treatment of patients with IVIg at the time of transplant is associated with a marked reduction of PFR-MCA hydrolyzing IgG 3 months post-transplant

Twenty-four sensitized patients (11 men and 13 women) had been treated with IVIg at the time of renal transplant. Treatment consisted in 4 cycles of administration of IVIg from the day of renal transplant and at day 21, 42 and 63 post-transplant. There was no difference between IVIg-treated patients and the remaining patients in terms of age, male to female sex ratio, dialysis time prior to transplant and HLA mismatch score (Table 1). In contrast, and as expected, 47.8% of the IVIg-treated patients had been transplanted previously as compared to 7.1% in the group of patients not treated with IVIg (P<0.0001). Likewise, 81.8% of the IVIg-treated patients had anti-HLA1 and anti-HLA2 antibodies, as compared to 48.2% for the remaining patients. Fifteen of the IVIg-treated patients received anti-thymocyte globulins and 8 received Basiliximab (one additional patient received both) (Table 1).

Patients treated and patients not treated with IVIg demonstrated similar rates of IgG-mediated PFR-MCA hydrolysis prior to renal transplant (6.1±1.0 vs 6.8±1.2 fmol/min/pmol, respectively, mean±SEM, Figure 1A). In contrast, the two groups of patients presented with statistically different rates of PFR-MCA hydrolysis by patients' IgG 3 months post-transplant: 1.5±0.3 fmol/min/pmol and 2.7±0.2 fmol/min/pmol for IVIg-treated and non-treated patients (P = 0.004, using the two-tailed Mann-Whitney test). While both groups of patients experienced a decrease in catalytic activity during the first 3 months post-transplant, the decrease was more marked among IVIg-treated patients than in patients who did not receive IVIg: 4.0 vs 2.5-folds reduction, respectively. Levels of catalytic IgG were not different between the two groups of patients 12 months (3.5±0.6 vs 3.0±0.3 fmol/min/pmol, respectively) and 24 months (5.4±1.1 vs 5.0±0.7 fmol/min/pmol, respectively) post-transplant. Except in the case of the use of IVIg, there was no difference in the levels of catalytic IgG according to the type of induction therapy. For instance, patients treated with anti-thymocyte globulins (ATG) presented with similar levels of catalytic IgG as patients who did not receive ATG (Figure 1C).

### Levels of PFR-MCA-hydrolyzing IgG are not associated with the presence of anti-HLA antibodies

**Figure 2:**
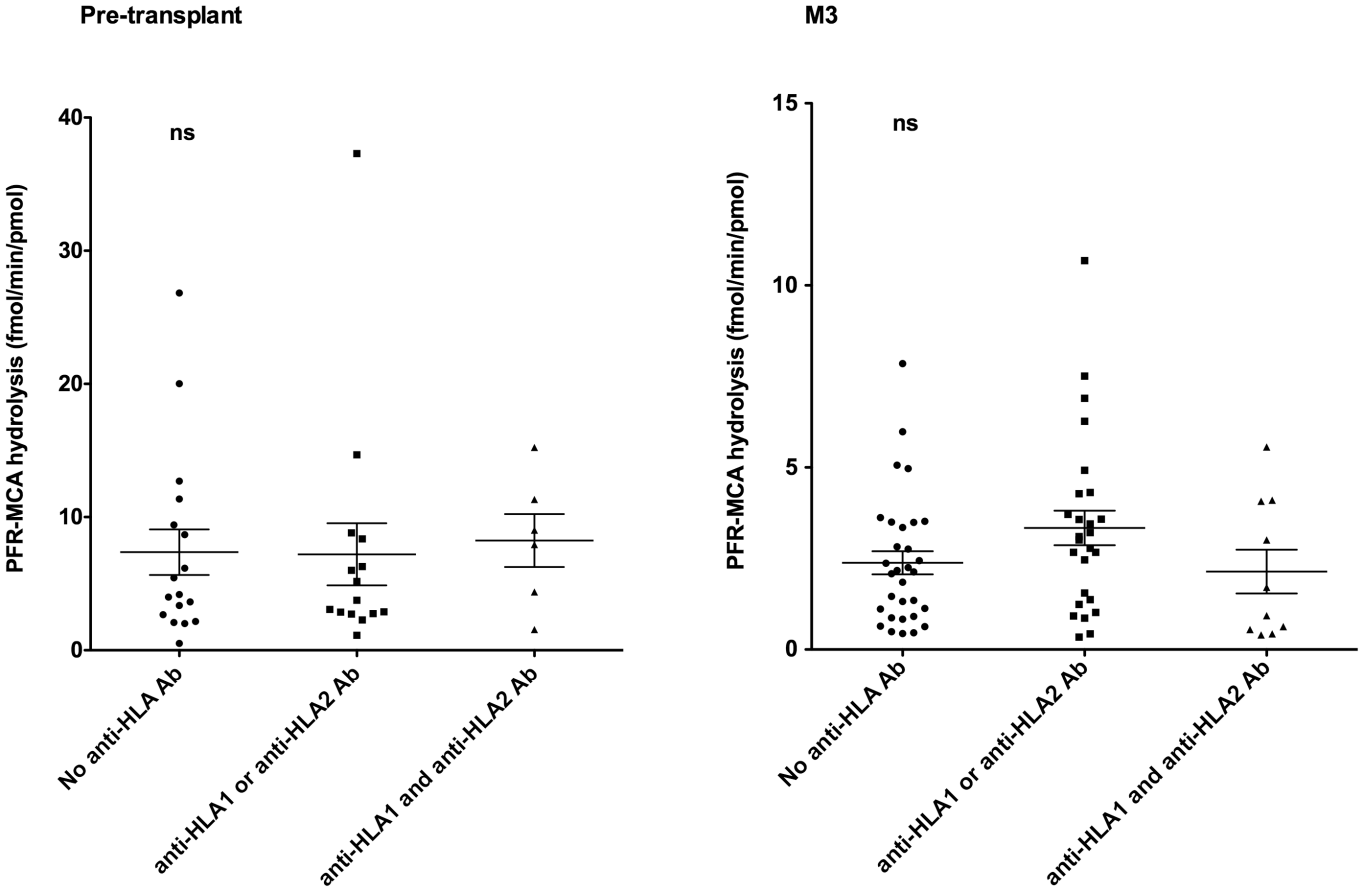
Levels of IgG-mediated hydrolysis of PFR-MCA among patients with anti-HLA antibodies. Patients were divided into three groups based on the presence of anti-HLA antibodies: no anti-HLA antibodies (circles); presence of either anti-HLA1 or anti-HLA2 antibodies (squares), and presence of both anti-HLA1 and anti-HLA2 antibodies (triangles). The graphs depict the rates of hydrolysis of PFR-MCA by IgG from each groups of patients purified from plasma collected prior to renal transplant (D0) or 3 months after transplant (M3).

Because IVIg are generally administered to patients with anti-HLA antibodies [30], we investigated a possible bias between patients treated or not with IVIg with respect to the levels of PFR-MCA-hydrolyzing IgG. Patients were categorized based on the presence of either anti-HLA1 or anti-HLA2 antibodies, of both anti-HLA1 and anti-HLA2 antibodies or on the absence of anti-HLA antibodies. As is depicted in Figure 2, levels of PFR-MCA-hydrolyzing IgG were identical, irrespective of the presence or absence of anti-HLA1 and/or anti-HLA2 antibodies. This was true both prior to renal transplant and 3 months after transplant.

## Discussion

The pathophysiological role of catalytic antibodies in the human is yet unclear. We and others had initially reported the presence of naturally occurring catalytic antibodies in human immunological and inflammatory diseases [8], [10], [11], [31], leading to the hypothesis that these antibodies may be deleterious. However, the description of catalytic IgM, IgG and IgA under physiological conditions [13], [14], [15], the finding that levels of PFR-MCA hydrolyzing IgG were elevated in patients surviving from septic shock [21], and the discovery in some patients with acquired hemophilia, a disease characterized by the presence of neutralizing anti-factor VIII autoantibodies, of factor IX-activating IgG [32], encouraged to revisit the initial hypothesis. Notably, we demonstrated in renal-transplanted patients that patients who did not develop CAN two years after transplantation had more elevated levels of PFR-MCA-hydrolyzing IgG [22]. Our data highlighted the predictive value of measuring levels of catalytic IgG on the occurrence of CAN. The present work shows that patients with renal failure exhibit higher level of catalytic IgG than pooled normal IgG from healthy individuals (pre-transplantation data, Figure 1). It also reveals an extreme heterogeneity among patients in terms of level of PFR-MCA-hydrolyzing IgG.

Our data document a reduction in the levels of catalytic antibodies after kidney transplant, followed by a progressive and slow restoration of the repertoire of catalytic IgG over time. Fluctuations in levels of catalytic IgG have rarely been studied earlier. Previous reports concentrated only on small numbers of patients in a retrospective manner, with low number of time points studied using serum or plasma that had not been systematically collected at pre-defined time points [33], [34]. In contrast, one of the strength of the present study is the collection of plasma from a large number of consecutive patients in a prospective manner, and at different pre-determined time points: 3, 12 and 24 months following kidney transplant. Only the pre-transplant plasma samples were retrieved retrospectively. Of note, PFR-MCA-hydrolyzing IgG were tested at a constant IgG concentration for all the patients' samples (i.e., 67 nM). Hence, the changes in levels of catalytic IgG represent changes of the amount of catalytic IgG within the total IgG pool, and are thus independent from possible fluctuations of total IgG levels. Reasons for a drop in the levels of PFR-MCA-hydrolyzing IgG are probably linked to the immuno-suppressive treatment of the patients, in particular in the induction phase where high dose of immunosuppressive drugs are used.

In our cohort, 24 of 100 patients received IVIg therapy at the time of kidney transplant because they were sensitized. IVIg was used, either alone or in conjunction with plasmapheresis and/or Rituximab. We observe here that the drop in the levels of PFR-MCA-hydrolyzing IgG 3 months following kidney transplant was statistically significantly more pronounced for patients treated with IVIg, as compared to patients who did not receive IVIg as adjunctive therapy. Mean levels of catalytic IgG in IVIg-treated patients were 55% that of the other patients. A reduction of “panel-reactive antibody” values following IVIg infusion to renal transplanted patients has been previously documented [30], [35], [36]. Although statistically significant in the studies by Jordan *et al*, the decrease in donor-specific antibodies among IVIg-treated patients was not as marked and consistent as the one we observe in the case of catalytic IgG.

Two explanations may account for the further reduction in levels of catalytic IgG 3 months post-transplant in IVIg-treated patients. The dose and timing of IVIg administration to the patients are compatible with a direct dilution effect of patients' IgG by the infused immunoglobulins. Indeed, considering that the patients received 2 g IVIg per kg body weight, that the average patient weight was 68 kg, that the blood volume may be estimated to approximately 5 liters, that the half-life of IVIg is 3 weeks (not considering the fact that some patients present with large proteinuria), and that the last administration of IVIg occurred 3 weeks prior to the 3-month sampling, one may broadly estimate a 2 to 3-fold dilution of the endogenous IgG by IVIg, which incidentally corresponds to the observed further 2-fold reduction in levels of catalytic IgG. Alternatively, IVIg are endowed with immuno-regulatory effects on several innate and adaptive immune cells. In particular, IVIg have been shown *in vitro* to impact on B-cell proliferation, survival and differentiation, as well as modulate immunoglobulin secretion [37], [38], [39], [40], [41], [42], [43]. Investigating putative changes in the levels of catalytic IgM and IgA that have been identified as proficient catalysts [14], [15], might be a strategy to delineate between the two possibilities.

Our earlier work performed in a retrospective study that implicated 20 patients with kidney transplant, described that higher levels of catalytic IgG, both 3 and 12 months post-transplantation, were associated with a reduced incidence of chronic allograft nephropathy. The receiver operating characteristic curve derived from the levels of IgG-mediated PFR-MCA hydrolysis indicated that the hydrolytic activity of circulating IgG was a potential predictive marker for CAN, superior to the widely used biological parameters such as glomerular filtration rate [44] or proteinuria [45]. The patients in the latter study had received similar immunosuppressive regimen as the patients included in the present cohort. Taken together, our data suggest that the reduction, associated with IVIg administration, in the levels of catalytic IgG in the patients 3 months post-transplant may jeopardize the use of catalytic antibodies as prognosis markers for chronic allograft nephropathy.
